# Deep learning in detection of osteoporosis: insights from recent evidence, and future directions

**DOI:** 10.1097/MS9.0000000000004316

**Published:** 2025-11-14

**Authors:** Saad Arsalan Wasti, Areesha Wasti, Durjoy Acharjee, Hrithick Acharjee

**Affiliations:** a Department of Medicine, King Edward Medical University, Lahore, Pakistan; b Department of Medicine, Continental Medical College, Lahore, Pakistan; c Department of Medicine, Dhaka Medical College and Hospital, Dhaka, Bangladesh; d Department of Medicine, Sylhet M.A.G. Osmani Medical College, Sylhet, Bangladesh

*To the Editor*,

Osteoporosis is a silent but serious public health concern. It is a common disease involving systemic impairment of bone mass and microarchitecture, resulting in fragility fractures^[1]^. These fragility fractures drive morbidity, mortality and healthcare cost, yet the disease is commonly diagnosed only after fracture. Current diagnostic modalities suffer from limitations, including radiation exposure and limited access, and thus remain underutilized^[2]^. Recent work is exploring whether deep learning (DL) can utilize existing routine imagings [such as chest X-ray, computed tomography (CT), spine/hip radiographs, and panoramic dental films] to detect osteoporosis without having people face additional radiation dosages or access-restraints in diagnosing osteoporosis.

A recent hybrid DL model has shown a high accuracy of 86%, with similarly high specificity and precision in detecting osteoporosis using knee X-rays^[3]^. Another DL model for opportunistic osteoporosis screening from chest radiographs reported promising results across multiple settings, exceeding tests such as calcaneal quantitative ultrasound in performance on external data^[4]^. DL convolutional neural network models to unenhanced chest CT also showed good results in predicting osteoporosis. The Densenet121 model showed area under the curves greater than 0.8 in external test sets^[5]^.

In addition, cervical radiograph studies indicated the algorithm’s diagnostic accuracy, sensitivity, and specificity, and DL’s rate of corrected answers were considerably higher than spine surgeons (80.0% compared to 60.6%). The diagnostic yield, too, was higher than that of the spine surgeons^[6]^. Magnetic resonance imaging DL studies also suggested clinical use, expanding DL’s anatomic scope beyond the traditional hip and knee imagings^[7]^.

Meta-analyses have also combined available evidence assessing the diagnostic accuracy of DL model–based osteoporosis prediction. A recent meta-analysis synthesizing literature up to 2023 included six studies and reported a sensitivity and specificity of 81% and 87%, respectively, which were indicative of good performance^[8]^. It concluded that DL was effective in extracting bone density information from plain radiographs, and suggested its possible clinical utility in opportunistic screening.

The clinical integration of DL models, however, still poses numerous challenges. Four interdependent aspects need extensive addressing. First, greater external validation and calibration is needed. A model that performs on a single-center test set may fail on different populations (age mix, scanner manufacturer, imaging protocols). Second, cost-effectiveness should be carefully considered to see whether integrating DL into clinical algorithms can provide meaningful advantages over existing diagnostic methodologies. Third, more studies outside of Asia should be conducted, as most researches and datasets implementing DL’s utility for osteoporosis come from Asian settings. Lastly, regulatory, explainability, and equity concerns must be addressed. For clinical deployment, models must be transparent about uncertainty, avoid amplifying population biases, and provide human-interpretable cues to help radiologists and primary care decide management and follow-up.

To conclude, DL may best be viewed today as a decision-support and case-finding adjunct rather than a stand-alone tool that can increase the diagnostic accuracy of osteoporosis if embedded into clinical pathways. DL has moved beyond proof-of-concept and is increasingly being tested against gold standard modalities to explore clinical value. The next generation of studies must therefore shift from stand-alone accuracy reports to trials and implementation evaluations that show whether opportunistic DL screening reduces fractures, is cost-effective, and is equitable across patient groups. Dataset diversification and enrichment, and assessing the impact of DL-based screening on long-term patient outcomes, including fracture prevention and quality of life, should be key priorities for future researches.

This letter adhered to the transparency in the reporting of artificial intelligence (TITAN) guidelines 2025^[9]^.
